# Advances in neuroimaging in cancer-related cognitive impairment

**DOI:** 10.3389/fnhum.2026.1738024

**Published:** 2026-01-21

**Authors:** Jinxin Li, Feiyun Cui, Yuanshan Yang, Qingting Zhang, Lijiao Zeng, Yulun Li, Yunxian Zhang, Jinbai Huang, Wei Wang

**Affiliations:** 1 Nuclear Medicine Department, The First Affiliated Hospital of Yangtze University, Jingzhou, Hubei, China; 2 Department of Medical Imaging, Health Science Center, Yangtze University, Jingzhou, Hubei, China; 3 Department of Radiology, The First Affiliated Hospital of Yangtze University, Jingzhou, Hubei, China; 4 Department of Rehabilitation Radiology, Beijing Rehabilitation Hospital, Capital Medical University, Beijing, China

**Keywords:** brain function, CRCI, malignant tumors, neuroimaging, radiotherapy

## Abstract

Cancer-related cognitive impairment (CRCI) is a cognitive dysfunction of the brain caused by the tumor itself and antitumor treatments such as radiotherapy, chemotherapy, endocrine therapy, and surgery. As a common complication of cancer, CRCI significantly affects patients’ quality of life. In recent years, the neurobiological mechanisms of CRCI have garnered widespread attention. Research indicates that cancer-related therapies lead to CRCI by affecting brain structure, function, metabolism, and blood perfusion. Various neuroimaging techniques, including magnetic resonance imaging (MRI), positron emission tomography (PET), and electroencephalography (EEG), have been extensively employed to investigate the neurobiological underpinnings of CRCI. This article reviews recent advancements in neuroimaging research on CRCI, focusing on its influencing factors and the neural mechanisms underlying different cognitive domains, and summarizes findings from relevant animal model studies.

## 1 Introduction

Cancer ranks as the second leading cause of death in the United States and remains the primary cause of death among individuals under the age of 85. The most recent cancer statistics from 2024 reported approximately 2,001,140 new cancer cases and 611,720 cancer-related deaths in the United States (Bray et al., 2024). Studies have reported that, in addition to gastrointestinal disturbances, liver and kidney toxicity, and drug-related allergic reactions, patients with non-central nervous system tumors may also experience neurocognitive decline following cancer therapy. Notably, some patients exhibit cognitive deficits before treatment initiation (Lv et al., 2020; Mayo et al., 2021; Parsons and Dietrich, 2019). Most recently, CRCI has been recognized as a condition caused by both cancer itself and cancer-related treatments. It is primarily characterized by impairments in cognitive abilities such as memory, attention, executive function, etc. These deficits significantly affect the patient’s daily life, interpersonal relationships, and overall quality of life (Horowitz et al., 2019; Lange et al., 2019; Morgans et al., 2021). Research suggests that the manifestation of CRCI is likely influenced by a complex interplay of multiple factors, including the treatment regimen, cumulative drug dosage, patient age, genetic background, psychological state, and the tumor itself. Different cognitive domains may correspond to distinct patterns of neural circuit impairment. Concurrently, preclinical animal models of CRCI play an indispensable bridging role in elucidating molecular and cellular mechanisms and testing potential interventions.

**Figure 1:**
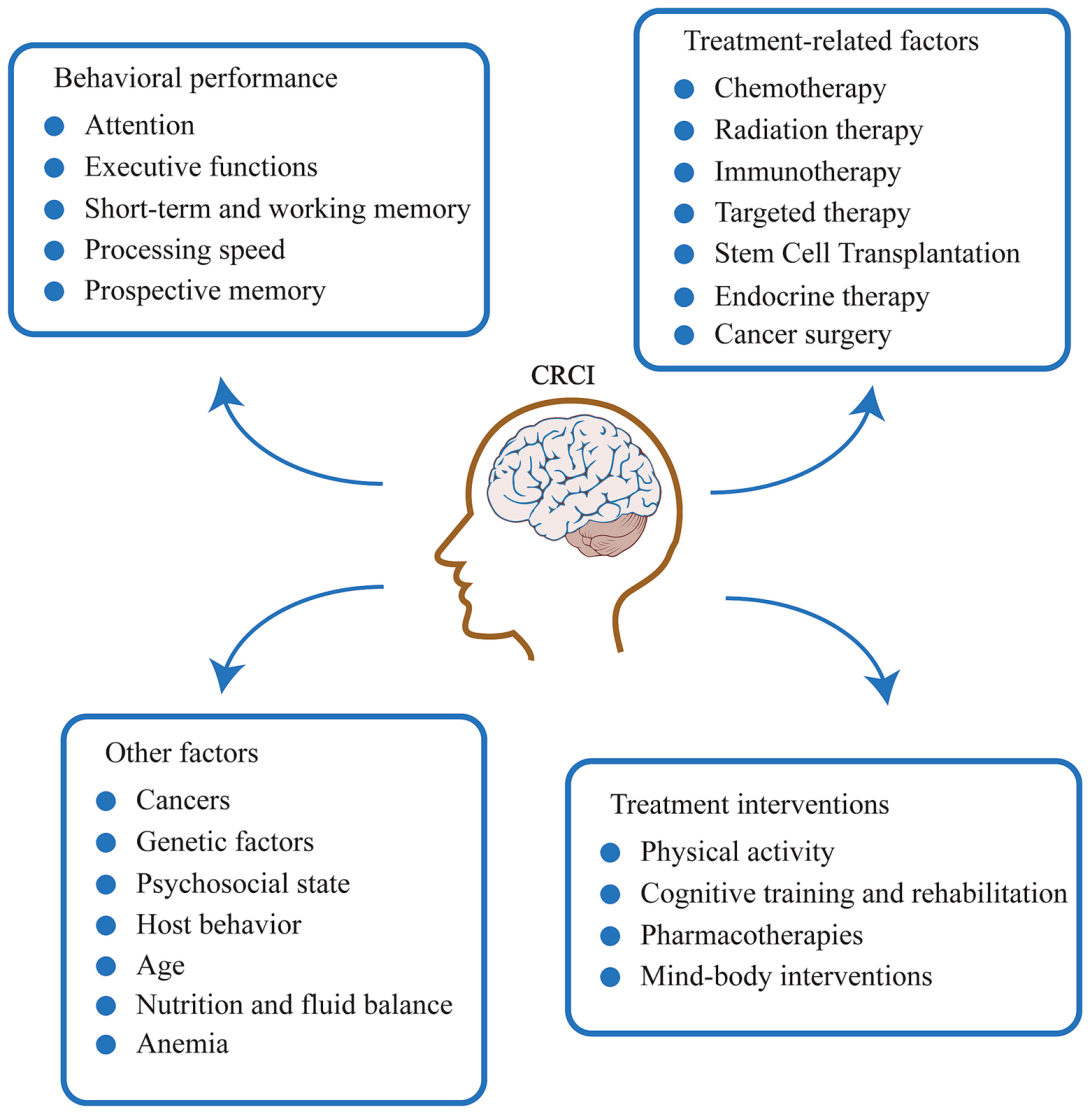
Clinical presentation, influencing factors, and intervention modalities of CRCI.

This article aims to review the current research progress in the field of CRCI neuroimaging. We will delineate how different treatment modalities and related factors affect the brain through specific pathways, summarize corresponding neuroimaging evidence by cognitive domain, and discuss the value and challenges of animal models in mechanistic research. This review seeks to provide a reference for a deeper understanding of the neural basis of CRCI, the development of objective biomarkers, and the formulation of effective intervention strategies (Figure 1 and Table 1).

**Table 1:** Summary of studies on neuroimaging techniques for CRCI across various cancer types.

Tumor type	Treatment	Cognitive assessment	Neuroimaging	Time	Key findings	The affected cognitive domains	Were the results independently replicated?	References
BC	Doxorubicin Paclitaxel	MMSE VFT DS	sMRI	After chemotherapy	Decreased gray matter density was observed in bilateral frontal lobes, right fusiform gyrus and bilateral cerebellum.mechanisms in BC patients	Overall cognitive function, verbal fluency and working memory/attention	The decrease in gray matter density after chemotherapy has been replicated in multiple studies.	Li et al. (2018)
BC	Adjuvant HT but not chemotherapy	FACT-Cog CES-D STAI	sMRI fMRI	Before treatment, 6 months after treatment, 1 year after treatment	Reduced gray matter volume in the frontal, temporal and parietal lobes and decreased cortical thickness in the prefrontal, parietal and insular cortices.	Attention, processing speed and executive function	The association between hormone therapy and brain structure changes has been reported multiple times.	McDonald et al. (2022)
BC	3FEC-3 T 4EC-4 T 4TC 6TEC	AVLT FACT-Cog	fMRI sMRI	Before chemotherapy, after chemotherapy	Increased ReHo in the right orbitofrontal region and the left dorsolateral prefrontal cortex; decreased ReHo in the cerebellum and the right middle/superior temporal gyrus; alterations in white matter integrity.	Memory	Part (The ReHo alteration patterns are unique, but WM damage is widely replicated)	Bai et al. (2021)
BC	MBI PT WL	Visual-verbal n-back task	fMRI	Before intervention, after intervention and 3 months after intervention	The activation in the right middle frontal gyrus and angular gyrus was decreased, while the activation in the right posterior cingulate gyrus was increased.	Working memory	Still to be verified (mechanistic intervention studies, more repetitions needed)	Melis et al. (2023)
BC	Doxorubicin Cyclophosphamide Docetaxel	MoCA HAMD AVLT ANT	ASL-MRI	Before chemotherapy, after chemotherapy	Increased perfusion in brain regions of the alertness and executive control network	Attention, memory and executive function	This has been supported by PET-CBF studies after chemotherapy	Chen et al. (2017)
Multiple cancers	Methotrexate	WRAML DKEFS	^18^F-FDG PET/MRI	One to 6 years after treatment	The SUVmean and CBFmean of the prefrontal cortex and cingulate gyrus decreased.	Memory and executive function	This study is the first PET/MRI multimodal report and needs to be verified in similar populations	Baratto et al. (2024)
DLBCL	The CHOP chemotherapy regimen	None	^18^F-FDG PET/CT	After chemotherapy	Increased metabolism in the bilateral hippocampus and parahippocampal gyrus, as well as decreased metabolism in the left medial orbitofrontal gyrus and superior frontal gyrus.	Not directly evaluated	Changes in brain metabolism after chemotherapy have been replicated, but the specific patterns in certain brain regions need to be verified	Hu et al. (2022)
NHL	Prophylactic intrathecal chemotherapy	None	^18^F-FDG PET/CT	Before chemotherapy and after chemotherapy (median 456 days)	Metabolism in the parietal lobe and cingulate gyrus increases, while metabolism in deep gray matter nuclei and the brainstem decreases.	Not directly evaluated	It is confirmed that chemotherapy can cause persistent and multi-brain-region metabolic changes.	Shrot et al. (2019)
ALL	TIT HDMTX	CPT ANT	fMRI	After the treatment is over	Reduced activation was observed in the right temporal and bilateral frontal–parietal regions during the CPT task, while increased activation was noted in the ventral prefrontal cortex and other areas during the ANT alertness task.	Attention, alertness, and orientation functions	The alteration of task fMRI activation patterns after chemotherapy has been replicated in studies of breast cancer and others.	Fellah et al. (2019)
ALL	CRT	WAIS RCFT TMT	sMRI	A median of 34 years after diagnosis	Microstructural alterations in the fornix, uncinate fasciculus, and ventral anterior cingulate white matter (decreased FA and MK)	Memory, visual–spatial function, executive function, attention, processing speed	Specific white matter tract damage in ALL survivors has been reported multiple times.	Follin et al. (2019)
ALL	Chemotherapy	None	sMRI	Long-term survivors after drug withdrawal	Reduced gray matter volume in the lingual gyrus, left middle occipital/middle temporal gyrus, etc.; decreased white matter FA and AD, increased MD and RD.	Not directly evaluated	Extensive changes in brain structure after chemotherapy, including reduced gray matter volume and decreased white matter integrity, have been repeatedly observed in multiple studies.	Zou et al. (2017)
LC	Platinum-based chemotherapy	TMT BDI MDRS	sMRI	Before chemotherapy (NSCLC), 1 month after chemotherapy (SCLC)	Before chemotherapy, the gray matter density in multiple brain regions of NSCLC patients decreased; after chemotherapy, the gray matter density in the hippocampus and other areas of SCLC patients decreased.	Language memory impairment, visual–spatial and language fluency	Structural brain abnormalities are present in lung cancer patients before treatment, and it is widely supported that key regions such as the hippocampus are affected after chemotherapy.	Simó et al. (2015)
LC	None	CFQ BDI STAI	sMRI	Before chemotherapy	Reduced global and local efficiency of the white matter network	Subjective cognitive failure, emotion	Abnormalities in brain networks before cancer treatment have been reported, but more validation is needed for changes in topological properties.	Liu et al. (2022)
LC	Platinum chemotherapy	None	MRI	After chemotherapy	The thickness of the frontal lobe, temporal lobe and insular cortex is reduced.	Not directly evaluated	The association between platinum-based chemotherapy and cortical thinning has been observed in multiple studies.	Lv et al. (2022)
LC	None	None	^18^F-FDG PET/CT	Before chemotherapy	Metabolism is elevated in the thalamus, putamen, etc., while it is decreased in the inferior parietal lobule, fusiform gyrus, etc.	Not directly evaluated	Abnormal brain metabolism in non-CNS cancer patients before treatment has been repeatedly demonstrated by multiple PET studies.	Zhang et al. (2016)
LC	Chemotherapy	None	^18^F-FDG PET/CT	During chemotherapy (after 2–4 cycles) and 6 months after the end of chemotherapy	Persistent metabolic abnormalities during chemotherapy; 6 months after the end: Abnormalities tend to recover.	Not directly evaluated	The dynamic patterns of brain metabolic changes during chemotherapy and partial recovery after treatment have been described.	Yu et al. (2023)
CRC	None	None	fMRI	Before treatment	The ReHo/ALFF/DC values in multiple brain regions such as the postcentral gyrus, middle occipital gyrus, and lingual gyrus decreased.	Not directly evaluated	Cancer itself can cause changes in brain function activities, but the specific brain region patterns need to be verified in a larger sample of CRCI.	Xu et al. (2024)
CRC	Chemotherapy	MMSE FACT-Cog	fMRI	After chemotherapy	The fALFF in the left anterior cingulate gyrus and middle frontal gyrus decreased; the fALFF in the left superior frontal gyrus (orbital part) and middle occipital gyrus increased.	Overall cognitive function, subjective cognitive function	To be verified	Liu et al. (2022)
CRC	CTX	TMT AVLT GDS	ERP fMRI	After chemotherapy	The DAN node activity increased, accompanied by focal gray matter volume reduction; ERP P3 amplitude alteration.	Attention, processing speed, memory, executive function, emotion	It provides the correlation between electrophysiology, imaging and behavior, with a unique pattern that requires independent replication.	Berger et al. (2023)
PCA	Intermittent ADT	SOPT POMS The Stroop test	^18^F-FDG PET/CT	Before ADT treatment and 9 months after ADT treatment	Examine changes in brain metabolism using PET in men undergoing	Spatial working memory, verbal memory, emotion, executive function	The finding that ADT leads to reduced metabolism in brain regions such as the posterior cingulate gyrus has been supported by subsequent studies.	Cherrier et al. (2018)
PCA	ADT	QoL N-back task	sMRI	Before ADT treatment and 6 months after ADT treatment	The thickness of the frontal pole cortex increases.	Working memory, quality of life	Early cortical thickening in ADT has been reported, which is different from the traditional “atrophy” hypothesis and requires more research for verification.	Chaudhary et al. (2024)

BC, breast cancer; HC, healthy controls; MMSE, mini-mental state examination; VFT, verbal Fluency Test; DS, Digit Span; HT, hormonal therapy; FACT-Cog, functional assessment of cancer therapy-cognitive function; CES-D, center for epidemiologic studies-depression scale; STAI, state–trait anxiety inventory; 3FEC-3 T, (5-fluorouracil + epirubicin + cyclophosphamide) × 3paclitaxel × 3; 4EC-4 T, (Epirubicin + Cyclophosphamide) × 4-Paclitaxel × 4; 4TC, Docetaxel × 4; 6TEC, Paclitaxel + Epirubicin + Cyclophosphamide; AVLT, auditory verbal learning test; MBI, mindfulness; PT, physical training; WL, waitlist control condition; MoCA, the montreal cognitive assessment; HAMD, the hamilton depression rating scale; WRAML, wide range assessment of memory and learning; DKEFS, delis-kaplan executive function system; DLBCL, diffuse large B-cell lymphoma; NHL, non-hodgkin lymphoma; ALL, Acute Lymphoblastic Leukemia; CPT, the continuous performance task; ANT, the attention network task; TIT, triple intrathecal treatments; HDMTX, high-dose methotrexate; CRT, cranial radiotherapy; WAIS, wechsler adult intelligence scale; RCFT, rey complex figure Test; TMT, trail making test; LC:lung cancer; SCLC, small-cell lung cancer; NSCLC, non-small-cell lung cancer; BDI, beck depression inventory; MDRS, mattis dementia rating scale; CFQ, cognitive failure questionnaire; TBSS, tract-based spatial statistics; CRC, colorectal cancer; CTX, Cyclophosphamide; GDS, global deficit scores; ADT, androgen deprivation therapy; PCA, prostate cancer; SOPT, self-ordered pointing test; POMS, the profile of mood states; QoL, quality of life.

## 2 Search strategy and criteria

**Figure 2:**
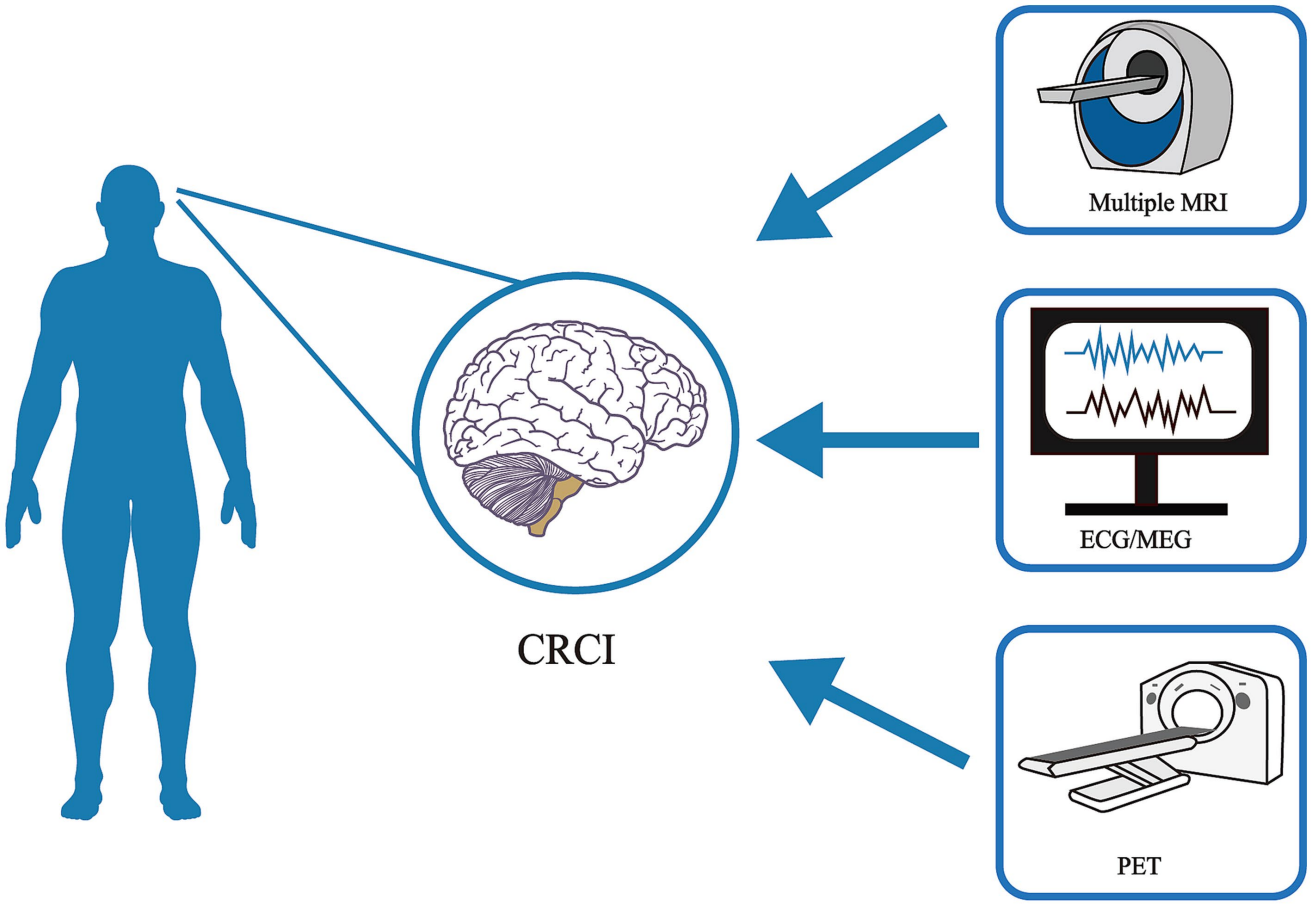
Common neuroimaging techniques for CRCI.

This review was constructed by searching relevant literature in the PubMed database from 2012 to 2024. The search strategy employed keywords such as “CRCI,” “cancer,” “PET,” “MRI,” “chemotherapy,” and “radiotherapy.” For animal model studies, searches were conducted by combining keywords like “cancer” with “brain” or “central nervous system,” and “cognition” with “animal” or “mouse” or “rat.” During literature screening, duplicates were excluded, and studies not aligned with the focus of this review were filtered out. Two reviewers independently screened the full texts, titles, and abstracts. Ultimately, only representative studies were included (Figure 2).

## 3 Factors influencing CRCI

### 3.1 Neurotoxicity of treatment regimens and dosage

The pathways through which chemotherapeutic agents induce cognitive dysfunction are related to their differential effects on the blood–brain barrier, which in turn leads to varied neuroimaging manifestations. Studies indicate that patients treated with anthracycline-containing regimens show significantly elevated serum levels of pro-inflammatory cytokines such as TNF-*α* and IL-6. The levels of these inflammatory markers are significantly associated with increased subjective cognitive complaints and declines in objective neuropsychological test performance (Cheung et al., 2015; Janelsins et al., 2017).

Platinum-based agents exhibit different properties. The choroid plexus, a key component of the blood-cerebrospinal fluid barrier and the primary site of CSF production, may be involved. Platinum drugs are known primary etiological agents for peripheral neuropathy, with mechanisms potentially involving direct damage to dorsal root ganglia. This damage suggests that platinum drugs may more directly affect the central nervous system microenvironment, subsequently impacting adjacent brain regions such as the hippocampus. Research by Simó et al. demonstrated decreased gray matter density in multiple brain regions, including temporolimbic structures like the hippocampus and parahippocampal gyrus crucial for cognitive function, in lung cancer patients receiving platinum-based chemotherapy (Cheung et al., 2015). This finding directly links drug characteristics to observable structural changes in the human brain.

Studies show that a higher number of courses and doses of methotrexate treatment leads to a decreased prevalence of leukoencephalopathy in acute lymphoblastic leukemia (ALL) survivors after one and a half years (Reddick et al., 2005). CSF analysis suggests that long-term elevation of axonal injury markers like myelin basic protein (MBP) in survivors provides molecular-level support for persistent white matter damage (Cheung et al., 2018). In neuroimaging, this microstructural damage manifests as alterations in diffusion tensor imaging (DTI) parameters. Zou et al. (2017) observed decreased fractional anisotropy (FA) and increased radial diffusivity (RD) in the white matter of ALL survivors who received chemotherapy. Notably, these alterations in white matter integrity and associated cognitive difficulties remain detectable years after treatment cessation, indicating potentially long-term effects. However, studies by Baratto and Shai et al. suggest that high-dose methotrexate in hematologic malignancies like lymphoma may induce more diffuse white matter damage, appearing as reduced glucose metabolism and blood flow in specific brain regions on imaging. They demonstrated that the mean standardized uptake value (SUVmean) and mean cerebral blood flow (CBFmean) in the prefrontal cortex and cingulate gyrus could quantitatively detect executive cognitive function (Baratto et al., 2024; Shrot et al., 2019). Specific results require further analysis.

Prostate cancer has surpassed lung cancer as the most common cancer in men globally and a leading cause of male cancer death (Bray et al., 2024). Research indicates that cognitive dysfunction in male reproductive system cancer patients may be associated with significantly reduced testosterone levels. Testosterone exerts a protective effect against cognitive dysfunction primarily mediated through androgen receptors, involving free radical scavenging and enhanced synaptic plasticity. This is mainly observed in prostate cancer patients undergoing androgen deprivation therapy (ADT) (Holtfrerich et al., 2020; Yan et al., 2019). ADT is a common treatment for patients with localized prostate cancer or those with a rising PSA after definitive therapy without evidence of metastasis. The frontopolar cortex (FPC) is considered crucial for working memory and other cognitive processes, including planning and managing multiple behavioral goals. Chaudhary et al. (2024) found that patients receiving 6 months of ADT exhibited increased cortical thickness (CT) in the FPC. Changes in testosterone levels were correlated with changes in FPC thickness and working memory across all participants.

### 3.2 Psychological factors

Multiple studies have found associations between cognitive impairment and anxiety, depression, sleep disturbances, and post-traumatic stress (Boscher et al., 2020; Dhillon et al., 2018; Ng et al., 2018). Furthermore, the immense psychological stress following a cancer diagnosis can lead to declines in both subjective patient evaluations and neuropsychological test performance. Although specific neuroimaging markers for these symptoms are still under exploration, they should be controlled as covariates during analysis to distinguish emotion-related from treatment-specific brain alterations.

### 3.3 Age and genetics

Beyond psychological factors, age may also contribute to cognitive decline. Cancer might accelerate normal aging due to increased DNA damage and reduced repair capacity, limiting cognitive reserve and brain reorganization in cancer patients (Mandelblatt et al., 2013). Studies show that compared to untreated patients and controls, older breast cancer patients receiving chemotherapy had poorer baseline cognitive reserve and more severe CRCI, particularly in processing speed (Ahles et al., 2010). Additionally, age-related declines in hormone levels have been found to play a role in CRCI, as anti-hormone therapies can amplify cognitive dysfunction induced by hormonal fluctuations in elderly cancer patients (Harrison et al., 2021). In neuroimaging analysis, age-related brain changes, such as natural hippocampal volume reduction, are strong confounding factors that must be statistically controlled; otherwise, they may mask or exaggerate treatment-specific effects.

Genetics also contribute to a patient’s risk of developing CRCI. Research indicates that genes for catechol-O-methyltransferase (COMT), apolipoprotein E (APOE), and brain-derived neurotrophic factor (BDNF) are closely linked to the occurrence of CRCI. Individuals with these specific genetic variants appear more susceptible to CRCI (Gonzalez et al., 2015; Kautiainen et al., 2023).

## 4 Neuroimaging research Progress in CRCI by cognitive domain

### 4.1 Attention

Attention is fundamental to cognitive function. Multiple studies report that cancer treatment, particularly chemotherapy, is associated with subjective complaints and objective test performance declines in attention among patients. Neuropsychological studies show impaired attention in breast cancer patients on tasks like the Continuous Performance Test (CPT) post-chemotherapy. Event-related potential (ERP) studies observe reduced amplitude of the P3 (P300) component in these patients (Kam et al., 2016). EEGstudies also find abnormal brain electrical activity in chemotherapy patients (Moore et al., 2014). fMRIresearch indicates altered activation patterns in brain regions like the prefrontal cortex during working memory or attention tasks post-chemotherapy (McDonald et al., 2012). In pediatric ALL patients, Fellah et al. (2019) also found treatment-related alterations in brain activation patterns during attention tasks. Patient self-reported attention problems are common. A large-scale study found a significant increase in subjective cognitive complaints in breast cancer patients after chemotherapy (Janelsins et al., 2017). However, the correlation between subjective cognitive complaints and objective neuropsychological test results is often low, suggesting these methods may reflect different dimensions of cognitive impairment or be influenced by different factors. Kesler (2014) using resting-state fMRI, found altered functional connectivity in brain networks, with the default mode network (DMN) potentially serving as a biomarker in breast cancer patients post-chemotherapy. DTI studies consistently report decreased white matter microstructural integrity in breast cancer patients after chemotherapy, manifested as reduced FA, and this white matter damage correlates with declines in cognitive test performance for attention and processing speed (Deprez et al., 2011; Mzayek et al., 2021). Chen, using arterial spin labeling (ASL), found changes in cerebral blood flow (CBF) in attention network-related brain regions in breast cancer patients receiving neoadjuvant chemotherapy (Chen et al., 2017). Findings suggest that cognitive impairment patterns may differ across cancer types. Research has observed glucose metabolism abnormalities in attention-related brain regions like the thalamus in untreated lung cancer patients (Zhang et al., 2016). In contrast, cognitive impairment in breast cancer patients is often associated with specific chemotherapy regimens, such as those containing anthracyclines (Kesler and Blayney, 2016).

### 4.2 Executive function

Executive function encompasses cognitive flexibility, working memory, inhibitory control, and planning ability. It is one of the most commonly affected and functionally significant cognitive domains in CRCI. Neuroimaging studies indicate that executive dysfunction is closely associated with structural and functional abnormalities in the prefrontal cortex and its related networks (Lange et al., 2019). Research confirms that cancer treatment correlates with multi-dimensional impairment of executive function. Studies collecting test results like Digit Span and Verbal Fluency in breast cancer patients post-chemotherapy have demonstrated chemotherapy-related executive function impairment, linked to decreased gray matter density in prefrontal regions such as the right middle frontal gyrus (Li et al., 2018). Furthermore, prostate cancer patients receiving ADT show reduced capacity on the n-back task assessing working memory (Chaudhary et al., 2024). These behavioral deficits directly reflect patient-reported declines in quality of life and daily functioning difficulties.

sMRI studies provide crucial anatomical bases for executive function impairment. Voxel-based morphometry (VBM) analysis shows significant reductions in gray matter density in the dorsolateral and ventrolateral prefrontal cortices of breast cancer patients receiving chemotherapy, with this structural change correlating with cumulative chemotherapy dose (Li et al., 2018). fMRI further reveals abnormal neural activity patterns in the prefrontal cortex during working memory tasks in chemotherapy patients. Some studies observe task-related hyperactivation, suggesting a compensatory mechanism reflecting decreased neural processing efficiency, where the brain recruits additional resources to maintain cognitive performance (McDonald et al., 2012).

The execution of executive functions relies on circuitry involving the prefrontal cortex and subcortical structures (e.g., striatum, thalamus) and the coordination of large-scale brain networks. DTI data indicate significantly decreased white matter tract integrity in brain regions like the frontal, parietal, and occipital lobes post-chemotherapy in breast cancer patients, manifested as reduced FA. Moreover, the degree of this white matter damage significantly correlates with declines in performance on cognitive tests of attention, processing speed, and memory (Deprez et al., 2011). Rs-fMRIreveals long-term effects of chemotherapy on brain function at the network level. Studies find significantly weakened functional connectivity within the executive control network (ECN) in breast cancer patients after chemotherapy. This abnormal network connectivity change significantly correlates with poorer performance on neuropsychological tests of executive function (Wang et al., 2016). This finding suggests that functional dysregulation of large-scale brain networks is a key aspect in understanding the neural mechanisms of executive dysfunction. It provides potential neuroimaging biomarkers for future development of preventive cognitive interventions or adjustment of treatment strategies. Clarifying the specific neurotoxic pathways of different treatment modalities is crucial for developing targeted neuroprotective strategies and achieving personalized cognitive risk management.

### 4.3 Memory

Episodic memory is particularly impaired in CRCI. Multiple studies show declines in memory indices like delayed recall on neuropsychological tests such as the Auditory Verbal Learning Test (AVLT) in breast cancer patients receiving chemotherapy (Lange et al., 2019; McDonald et al., 2010). sMRI studies have found chemotherapy associated with reduced gray matter volume or density in several brain regions, including the hippocampus. As a core structure for episodic memory formation, structural alterations in the hippocampus are a significant reason for impaired memory encoding, consolidation, or retrieval in CRCI patients (Yao et al., 2023). Animal model research by Winocur et al. (2014) further supports this. Studies show that common chemotherapeutic agents like cyclophosphamide and doxorubicin inhibit hippocampal neurogenesis and correlate with deficits in animals on hippocampus-dependent memory tasks.

Working memory, as a temporary storage system for online information maintenance and manipulation, is also affected in CRCI (Lange et al., 2019). fMRI studies have revealed the neural basis of working memory impairment from the perspective of brain functional activity (McDonald et al., 2010). Research finds altered activation patterns in prefrontal and parietal brain regions associated with working memory when cancer patients perform tasks like the n-back task (Kesler, 2014). Some studies observe that patients need to recruit additional brain resources or exhibit different activation patterns under high cognitive load, which may indicate decreased neural processing efficiency—the brain working harder to maintain performance (Askren et al., 2014). Rs-fMRI studies also find altered functional connectivity within the frontoparietal network responsible for working memory, including connections between the prefrontal and posterior parietal cortices (Wang et al., 2016).

Kesler et al. (2013) using magnetic resonance spectroscopy (MRS), found changed metabolite concentrations in brain regions like the prefrontal cortex in breast cancer patients post-chemotherapy, with elevated choline and myo-inositol and a decreased N-acetylaspartate (NAA)-to-choline rati. These metabolic changes may reflect neuroinflammation, glial activation, or impaired neuronal/axonal integrity and correlate with patients’ subjective memory complaints. PET studies have shown altered glucose metabolism in memory-related brain regions like the posterior cingulate gyrus after ADT (Cherrier et al., 2018). Similarly, DTI shows chemotherapy can impair white matter microstructural integrity. Damage to white matter pathways connecting the hippocampus, such as the fornix and cingulum bundle, may directly disrupt neural circuits for memory information transfer (Follin et al., 2019).

The dynamic evolution of memory impairment presents a complex temporal pattern. Research indicates cognitive deficits may appear during or shortly after chemotherapy. While symptoms improve over time for some patients, cognitive issues persist long-term for others (Janelsins et al., 2017). Recovery potential may be influenced by various factors, including age, baseline cognitive function, and treatment regimen (Lange et al., 2019). Currently, research exploring CRCI management strategies is ongoing. Animal model studies suggest interventions like physical exercise may improve chemotherapy-induced memory deficits by promoting hippocampal neurogenesis and enhancing synaptic plasticity (Winocur et al., 2014). n clinical research, cognitive training and physical exercise have emerged as potential non-pharmacological interventions to help patients cope with cognitive difficulties and improve quality of life (Bray et al., 2017).

### 4.4 Information processing speed

Processing speed, a fundamental cognitive process, is commonly impaired in CRCI and likely broadly impacts other cognitive functions. Neuroimaging research provides important insights into this impairment, revealing underlying white matter microstructural alterations and brain network dysfunction (Lange et al., 2019). Patients often show performance declines on standardized neuropsychological tests assessing processing speed, such as Part A of the Trail Making Test and the Digit Symbol Substitution Test (Collins et al., 2014). From a network neuroscience perspective, efficient information processing relies on the overall coordination of brain structural and functional networks. Studies find altered topological properties of brain networks in CRCI patients. Specifically, global efficiency, measuring overall information transfer between different brain regions, tends to decrease. Concurrently, the optimal small-world network property—maintaining tight local connections while enabling efficient long-range communication between different brain regions—is weakened in CRCI patients, indicating their brain networks may deviate from the optimal pattern for information integration and processing (Amidi et al., 2017; Liu et al., 2022). These network-level changes imply less efficient and fluid information exchange between brain regions, explaining the slowing of processing speed.

### 4.5 Language function

Compared to memory and processing speed, language dysfunction has received relatively less attention in CRCI research, yet its impact may involve multiple levels from lexical retrieval to pragmatic communication. The Controlled Oral Word Association Test (COWA), included in the core assessment battery recommended by the International Cognition and Cancer Task Force (ICCTF), assesses verbal fluency, indicating lexical retrieval deficits are a measurable dimension of CRCI. Clinical observations and patient self-reports also note problems like word-finding difficulties and comprehension issues in complex situations, potentially affecting daily communication and quality of life (Deprez et al., 2018). Language dysfunction poses a potential threat to patients’ social interaction, occupational ability, and mental health, potentially exacerbating perceived cognitive decline and risk, especially in elderly patients (Lange et al., 2019). Currently, specific research on language impairment in CRCI remains insufficient. Future studies need to utilize multimodal neuroimaging techniques combined with refined language tasks to systematically elucidate its neural mechanisms and provide a basis for developing targeted rehabilitation strategies.

## 5 Neuroimaging studies in animal models of CRCI

In clinical settings, besides the tumor itself and cancer treatment, many factors may influence cognitive function, including comorbidities, age, cancer type, disease progression, differences in baseline cognitive testing, and treatment regimens. Establishing animal models of CRCI allows direct control of these variables (Demos-Davies et al., 2024). Since experimental animals are genetically identical, preclinical studies can control strain, sex, and environment to assess the neurotoxic effects of single cancer therapeutic agents, characterize the underlying mechanisms of cognitive deficits observed in cancer patients, and identify cognitive domains affected by cancer treatment (Matsos and Johnston, 2019). Currently, animal models have been used to study the impact of cancer treatment on cognitive function, with neuroimaging techniques employed for monitoring. Findings indicate that CRCI animal models are crucial for elucidating CRCI mechanisms and exploring treatments (Table 2).

**Table 2:** Animal models used for CRCI research.

Animal	No. of models	Treatment	Cognitive assessment	Neuroimaging	Main outcomes	References
APOE4 targeted-replacement C57BL/6 J mice	31	Single dose of 5 mg/kg IP DOX	Open Field Task, Elevated Zero Maze, Pre-Pulse Inhibition, Pre-Pulse Inhibition, Fear Conditioning.	VBM	Cognitive deficits observed in aged APOE4 knock-in mice following doxorubicin administration	Demby et al. (2020)
Female Sprague–Dawley rats	60	Single dose of 4 mg/kg IP DOX, 5 mg/kg IP donepezil	The Morris water maze test	^18^F-FDG PET/CT	Donepezil is clinically useful for addressing cognitive impairments that occur following chemotherapy	Lim et al. (2016)
Female Sprague–Dawley rats	18	Single dose of 1 mg/kg DOX via tail vein	Novel object recognition and Contextual fear conditioning	^18^F-FDG PET/CT, MRI	Impairment of prefrontal cortex may be one of the mechanisms underlying the occurrence of the DOX model of chemotherapy-induced cognitive dysfunction	Barry et al. (2018)
Female, BALB/c mice	36	Single dose of 0.75 mg/kg IP MTX, a single 16 Gy fraction of orthovoltage ionizing radiation	None	^18^F-FDG PET/CT	Radiation can trigger substantial brain bystander effects in areas remote from the directly irradiated cells and tissues	Feiock et al. (2016)
Female Long-Evans rats	38	37.5 mg/kg IP MTX + 50 mg/kg 5-FU (once a week)	Spatial memory, cued memory, non-matching to sample rule learning; delayed non-matching to sample rule learning	None	Exercise in preventing or treating cognitive impairment associated with chemotherapy is benefit	Winocur et al. (2014)
Wistar female rats	40	Every day 5 mg/kg cotinine	Novel location recognition test, Porsolt’s forced swim test, Rotarod	None	Treatment with cotinine may facilitate the recovery and diminish the cognitive consequences of chemotherapy	Iarkov et al. (2016)
Male Lister-hooded rats	48	A single dose of 10 mg/kg of fluoxetine	Novel location recognition	None	Fluoxetine can protect newly born hippocampal neurons from the cytotoxic effects of 5-FU	Lyons et al. (2012)

APOE, the apolipoprotein E; IP, intraperitoneal; DOX, doxorubicin; VBM, voxel-based morphometry; MTX, Methotrexate; 5-FU, 5-Fluorouracil.

### 5.1 Common animal models

Lim et al. (2016) found reduced glucose metabolism in the medial prefrontal cortex and hippocampus of rats treated with doxorubicin or cyclophosphamide. Barry et al. (2018) found decreased ^18^F-FDG uptake in the prefrontal cortex of rats 30 days after doxorubicin treatment, consistent with Lim’s findings. The doxorubicin/cyclophosphamide model simulates the anthracycline/alkylating agent combination chemotherapy commonly used in cancers like breast cancer. Animals receiving a single intraperitoneal injection of doxorubicin or cyclophosphamide showed significant impairment in episodic memory, spatial learning and memory, and contextual fear memory on behavioral tests like novel object recognition, Morris water maze, and fear conditioning. These behavioral phenotypes highly correspond to memory decline and executive dysfunction reported by clinical patients. Behavioral abnormalities occurred 1–4 weeks post-administration and could last months, simulating subacute cognitive impairment in the clinic. Winocur et al.’s (2014) rat antimetabolite model simulates treatments for colorectal cancer, lymphoma, and leukemia. Animals exhibited impairment in spatial working memory, reversal learning, and executive function, resembling the phenotypes of decreased processing speed and cognitive flexibility in clinical patients. Concurrently, mouse methotrexate models are used to simulate cognitive sequelae in pediatric ALL patients after high-dose methotrexate, showing long-term white matter damage and cognitive deficits (Melis et al., 2023). Cisplatin models can induce mitochondrial dysfunction and oxidative stress, with animals showing impaired performance on spatial memory and cognitive flexibility tasks, consistent with clinically observed cisplatin-related cognitive side effects (Lomeli et al., 2017).

### 5.2 Common models and their clinical relevance

Neuroimaging in animal models is primarily used for non-invasive monitoring of dynamic changes in brain structure and function, correlating with behavior. Findings show both overlap and differences with clinical studies.

Animal PET shows significantly reduced glucose metabolism in the medial prefrontal cortex and hippocampus of rats treated with doxorubicin or cyclophosphamide, consistent with clinical ^18^F-FDG PET results. These two brain regions are crucial for human cognition, especially executive function and episodic memory, making their hypometabolism a potentially cross-species consistent imaging marker for CRCI. Additionally, research confirms chemotherapy can inhibit neurogenesis in the hippocampal dentate gyrus (Christie et al., 2012; Dubois et al., 2014). This finding supports indirect clinical imaging observations of hippocampal volume reduction and altered functional connectivit (Feng et al., 2020)^,^ suggesting the hippocampus is a key target of chemotherapy neurotoxicity, and neurogenesis inhibition is a key mechanism in cognitive impairment. TSPO-PET studies suggest neuroinflammation involvement in CRCI (Schroyen et al., 2022)^,^ Animal models provide direct histological evidence that chemotherapy activates microglia, increasing pro-inflammatory cytokine expression in the hippocampus and prefrontal cortex, offering an explanation for clinically observed phenomena (Chiu et al., 2017).

However, while clinical DTI studies report widespread decreases in white matter microstructural integrity (Deprez et al., 2011; Menning et al., 2018)^,^ animal DTI models, although also showing white matter changes, present spatial patterns, severity, and associations with cognitive domains that are difficult to compare directly with human studies. This may be due to fundamental differences in white matter structure complexity, proportion, and function between rodents and humans. Secondly, fMRI studies reveal complex alterations in large-scale brain network connectivity in human (Kesler and Blayney, 2016; Wang et al., 2016). Although functional networks exist in rodents, their lower homology and complexity compared to humans limit direct study of these advanced brain network disorders and their association with specific cognitive symptoms in animal models.

An important feature of CRCI is that cognitive impairment persists for years or even decades in some patients, becoming a chronic sequela. Currently, animal models capable of simulating such long-term progressive impairment are scarce. One study used APOE4 gene-replaced mice treated with doxorubicin to simulate the interaction between genetic risk factors and chemotherapy, observing more persistent cognitive impairment and brain structural changes in aged mice (Demby et al., 2020)^,^ providing ideas for modeling high-risk subgroups. Overall, current models still have shortcomings in simulating the heterogeneity of human CRCI, its long-term dynamic evolution, and its comorbidities with the chronic disease state of cancer.

## 6 Discussion

A core and pervasive phenomenon in CRCI research is the inconsistency between patients’ subjective cognitive complaints and objective neuropsychological test results. A deep understanding of this inconsistency is crucial for elucidating the nature of CRCI and guiding clinical practice. Studies find that over 50% of breast cancer patients report cognitive problems after chemotherapy, but only about 15–25% show objective cognitive decline on standardized tests (Ahles et al., 2012). This discrepancy may arise from multiple mechanisms. Psychological and physiological factors, including states of anxiety, depression, fatigue, and insomnia, can significantly amplify patients’ perception of cognitive problems, while these factors have a relatively smaller impact on objective test performance (Dhillon et al., 2018). Therefore, subjective cognition largely reflects patients’ emotional distress and overall symptom burden. Neuroimaging research provides key information: even when patients perform normally on structured tests, their brains may have undergone compensatory changes. fMRI results show that patients may require additional brain region involvement or exhibit different activation patterns to maintain performance during cognitive tasks (Menning et al., 2017). This suggests that “normal” scores on objective tests may mask underlying decreased neural efficiency. More importantly, this inconsistency may reveal distinct neurocognitive phenotypes within CRCI itself. Just as Fan et al. (2023) identified subtypes in anxiety disorders characterized by impulsivity, each with markedly different brain structures, genetic risks, and clinical trajectories, similar subtypes may exist among CRCI patients. These subtypes exhibit varying sensitivities to neuropsychological testing, naturally leading to discrepancies between subjective and objective assessments. Consequently, multidimensional assessment is crucial for CRCI diagnosis, emphasizing the necessity of integrated evaluation using tools like the FACT-Cog scale that combine subjective reports and objective testing, with both being indispensable (Cheung et al., 2014). Subjective assessment reflects patients’ functional distress and quality of life impact, while objective testing provides a standardized measure of cognitive ability. Furthermore, assessment results should be interpreted considering individual baselines, educational background, and occupational demands. Even minor objective impairment can be significant for the individual. Most importantly, patients’ subjective cognitive complaints themselves are a valid and important clinical intervention target, regardless of accompanying objective impairment, as they directly relate to patients’ quality of life and functional status.

In a doxorubicin-induced rat CRCI model, administration of the cholinesterase inhibitor donepezil not only improved animals’ spatial learning and memory in the Morris water maze but also reversed the chemotherapy-induced reduction in glucose metabolism in the medial prefrontal cortex and hippocampus (Lim et al., 2016). This coupling of behavioral improvement and cerebral metabolic normalization provides strong preclinical evidence for CRCI treatment. In a 5-fluorouracil rat model, the selective serotonin reuptake inhibitor fluoxetine prevented chemotherapy-induced suppression of hippocampal neurogenesis and improved novel object recognition memory (Lyons et al., 2012), suggesting efficacy through protecting neural plasticity. Cotinine, the primary metabolite of nicotine, when administered post-chemotherapy to rats, showed improved memory and reduced depression-like behavior (Iarkov et al., 2016), with mechanisms potentially involving anti-inflammatory and neuroprotective effects. Both preventive and therapeutic interventions in animal studies show efficacy, suggesting a potentially large clinical intervention window, valuable from pre-treatment prevention to post-treatment phases. Different intervention strategies and targets should be selected for different timings.

## 7 Conclusion

This article systematically reviews advances in neuroimaging research on CRCI. Regarding influencing factors, different treatment regimens—such as anthracyclines, platinum-based agents, methotrexate, and ADT—produce specific neurotoxicity through pathways like inflammation, white matter damage, metabolic alterations, and hormonal fluctuations. Psychological and genetic factors also play significant roles. In terms of cognitive neural mechanisms, impairments in attention, executive function, memory, and processing speed are associated with structural abnormalities in brain regions like the prefrontal cortex and hippocampus, decreased white matter integrity, and altered functional connectivity in large-scale brain networks. Animal models partially replicate clinical phenotypes and key mechanisms but still face translational limitations. Future clinical trials should not merely aim to demonstrate improved cognitive test scores but also consider modulating specific pathophysiological pathways. For example, targeting neuroinflammation using TSPO-PET as a biomarker, or for neurogenesis disorder, exploring drug trials with serum or imaging biomarkers. Efforts must also be made to link macroscopic imaging changes with microscopic pathological processes. For example, alterations in cortical surface area and thickness may correspond to distinct cellular mechanisms (Kuang et al., 2023). Furthermore, drawing from animal research, future studies should concurrently assess behavior and combine it with imaging techniques, adopting a joint assessment approach that includes subjective reports, objective neuropsychological testing, and neuroimaging biomarkers. This is because imaging biomarker changes may indicate target engagement and early efficacy more sensitively and earlier than behavioral changes.
